# Der juvenile Granulosazelltumor – der Hodentumor der Kleinsten: Aktuelle Handlungsempfehlungen anhand von drei Kasuistiken

**DOI:** 10.1007/s00120-020-01391-7

**Published:** 2020-11-17

**Authors:** Maximilian Haider, Evi Comploj, Aybike Hofmann, Wolfgang H. Rösch

**Affiliations:** 1 Klinik für Kinderurologie in Kooperation mit der Universität Regensburg, Krankenhaus Barmherzige Brüder, Klinik St. Hedwig, Steinmetzstraße 1–3, 93049 Regensburg, Deutschland; 2 Klinik für Urologie, Caritas Krankenhaus St. Josef, Lehrstuhl für Urologie der Universität Regensburg, Regensburg, Deutschland; 3 Abteilung für Urologie, Zentralkrankenhaus Bozen, Bozen, Italien; 4 Landesfachhochschule für Gesundheitsberufe, Claudiana-Research, Bozen, Italien

**Keywords:** Hodentumor, Keimzell-Stroma-Tumoren, Hodensonographie, Pädiatrische Tumoren, Kinderurologie, Testicular cancer, Testicular sex cord stroma tumor, Scrotal ultrasound, Pediatric tumor, Pediatric urology

## Abstract

Der juvenile Granulosazelltumor (JGZT) des Hodens ist eine relevante Differentialdiagnose testikulärer Raumforderungen des Säuglingsalters. Es handelt sich um eine benigne Läsion, die Therapie ist rein chirurgisch. Metastasierungen oder Rezidive sind nicht bekannt. Am Beispiel von drei Kasuistiken wird das aktuelle diagnostische und therapeutische Management vorgestellt und diskutiert.

## Abstract (en)

Juvenile granulosa cell tumor of the testis is a relevant differential diagnosis regarding testicular neoplasia of the very young. This benign lesion requires surgical treatment. Metastases or recurrences have not been described in literature. We present three different cases and give recommendations for diagnosis and treatment.

## Fall A

**Figure 1:**
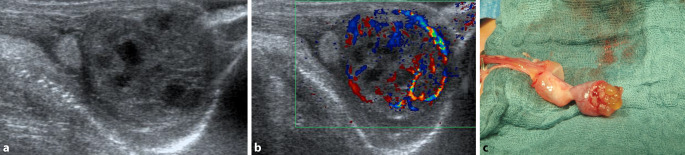

Ein männliches Neugeborenes (38 + 0 SSW, Geburtsgewicht 3600 g) wird wegen sekundären Stöhnens und Sauerstoffbedarf in die Kinderklinik verlegt. Nebenbefundlich fällt bei der Aufnahme ein prallharter linker Hoden auf. Labor: Blutbild unauffällig, Interleukin 6 erhöht, CRP zunächst im Normbereich. Im Röntgen-Thorax findet sich eine Flüssigkeitslunge. Unter dem Verdacht auf eine Neugeboreneninfektion Beginn einer parenteralen Antibiose. In der Hodensonographie (B-Bild) zeigt sich der linke Hoden orthotop mit multiplen intratestikulären Zysten im Bereich des Unterpols sowie dopplersonographisch Zeichen einer Hyperperfusion v. a. im Bereich der Hodenhüllen (Abb. 1a, b).

Bei insgesamt unklarem Befund mit nicht sicher auszuschließender peri-/postnataler Hodentorsion wird der linke Hoden notfallmäßig von inguinal operativ freigelegt. Der Hoden ist am Unterpol zystisch tumorös verändert mit multiplen, kleinen, gallertartig gefüllten Zysten ohne erkennbares Hodenparenchym (Abb. 1c). Am Hodenoberpol makroskopisch unauffälliges Hodengewebe. Der Unterpol kann in toto abpräpariert werden. Die Schnellschnittdiagnostik ergibt einen epithelialen zystischen Tumor unklarer Dignität, eine weitere Einordnung war anhand der Schnellschnitte nicht möglich. Der restliche Hoden wird daraufhin belassen und intraskrotal pexiert. Im weiteren Verlauf Beendigung der Antibiose bei unauffälligem Trachealsekret. Verlegung des Patienten am 4. postoperativen Tag auf Normalstation und Entlassung am 5. postoperativen Tag.

Die endgültige Histologie ergab aus dem intraoperativen Präparat einen Granulosazelltumor vom juvenilen Typ (JGZT). Die daraufhin veranlasste Chromosomenanalyse erbringt einen unauffälligen männlichen Chromosomensatz.

Im 4. Lebensmonat wird bei klinisch und laborchemisch (einschließlich Tumormarker) unauffälligen Befunden, aber sonographisch unregelmäßiger Parenchymtextur des verbliebenen Resthodengewebes eine skrotale Orchiektomie links durchgeführt. Histologisch bestätigt sich erneut der nun in toto resezierte juvenile Granulosazelltumor. Gleichzeitig findet eine Fertilitätsbeurteilung am Semidünnschnitt statt. Hier zeigen sich vorwiegend fetale A‑pale-Spermatogonien und nur sehr spärlich vom A‑dark-Typ (reife Formen).

Der Patient ist nun seit knapp 11 Jahren in Nachsorge ohne Hinweis für ein Rezidiv oder Metastasen.

## Fall B

Bei einem männlichen Neugeborenen im EU-Ausland wird unmittelbar postnatal eine Hydrocele testis diagnostiziert. Im Alter von 10 Wochen wird bei verändertem Tastbefund eine Sonographie durchgeführt, hier ergibt sich der Verdacht auf einen malignen Hodentumor. Im MRT-Becken mit Kontrastmittel erhärtet sich der Befund. Ein Hinweis auf eine Filialisierung besteht nicht. Mit 12 Wochen wird der Patient erstmals urologisch vorstellig. Bei der körperlichen Untersuchung findet sich eine schmerzlose, tastsuspekte Raumforderung des rechten Hodens. In der Sonographie ist das Parenchym des rechten Hodens nahezu vollständig durchsetzt von einer inhomogenen, gut durchbluteten, malignitätssuspekten und von den peritestikulären Strukturen abgrenzbaren Raumforderung. Die Tumormarker ergeben ein erhöhtes AFP mit 121,4 ng/ml (Norm im Alter >10 Monate: 3–28 ng/ml) bei normwertigem β‑HCG und LDH. Es erfolgt daraufhin die inguinale Freilegung. Intraoperativ kann der Tumor nur sonographisch vom spärlichen Hodenrest differenziert werden, deshalb Entschluss zur Orchiektomie. Der Patient wird am ersten postoperativen Tag beschwerdefrei entlassen.

Histologisch wird in der konventionellen Mikroskopie ein multizystischer juveniler Granulosazelltumor gesehen, dieser wird durch die Immunhistochemie und die spätere Referenzpathologie bestätigt.

## Fall C

**Figure 2:**
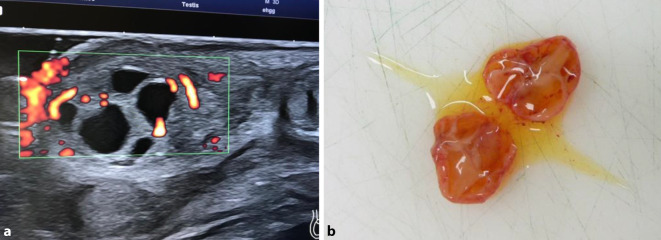

Ein Junge wird reif (39 + 2 SSW) mit Geburtsgewicht von 3530 g geboren. Im Rahmen der U2 fällt ein vergrößertes linkes Hemiskrotum mit palpatorisch verhärtetem Hoden auf. In der Sonographie (Abb. 2a) zeigen sich multiple zystische Veränderungen des Hodenparenchyms, die Abdomensonographie ist unauffällig. AFP 48.370 ng/ml, β‑HCG 0,3 U/l, LDH 428 U/l. Zu diesem Zeitpunkt wird der Verdacht auf einen JGZT gestellt. Am fünften Lebenstag erfolgt die inguinale Freilegung mit Tumorenukleation und Hodenerhalt links. Die Verdachtsdiagnose wird histologisch und später referenzpathologisch bestätigt. Entlassung am Folgetag. Vier Monate postoperativ zeigt sich ein klinisch und sonographisch unauffälliger Befund.

## Diskussion

**Figure 3:**
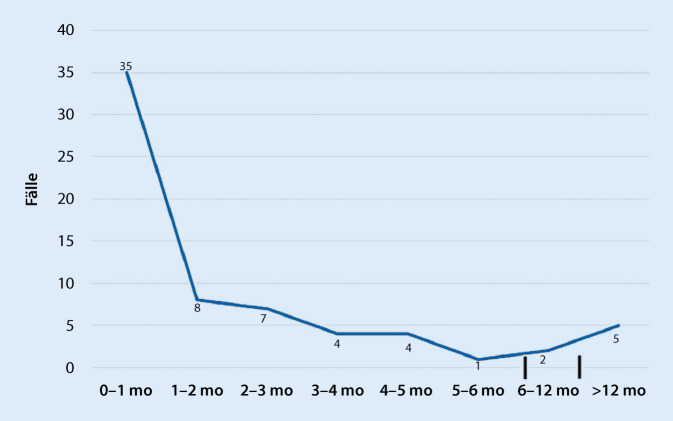

Der JGZT ist ein Tumor des Neugeborenen und des frühen Säuglingsalters. Damit nimmt er unter den kindlichen Tumorentitäten inklusive des Dottersacktumors eine Sonderrolle ein (Abb. 3; [15]). Gelegentlich kann die Diagnose bereits intrauterin gestellt werden [16].

Diagnostisch steht die Sonographie im Vordergrund. Bei passender Vorgeschichte und klinischem Bild ist durch den sonographischen Nachweis einer multizystischen, klar abgrenzbaren, intratestikulären Raumforderung das Vorliegen eines JGZT sehr wahrscheinlich. Gleichwohl ist die Diagnosestellung keineswegs trivial. Im Fall A wurde das Bild einer älteren Hodentorsion vorgetäuscht mit echoarmen, wabenartigen Strukturen, die durchaus auch hämorrhagischen Einblutungen oder bereits nekrotischen Arealen bei stattgehabter Torsion hätten entsprechen können. Im Fall B wurde der Sonographiebefund einem malignen Prozess zugeordnet. Differentialdiagnostisch muss neben anderen Tumorentitäten (z. B. Dottersacktumor) eine zystische Dysplasie des Rete testis erwogen werden [6]. Da sowohl Seminome als auch Chorionkarzinome vor der Pubertät praktisch nie auftreten, hat β‑HCG als Tumormarker im Kindesalter keine Bedeutung. Die Interpretation des Hodentumormarkers AFP ist aufgrund der physiologisch hohen Werte im frühen Säuglingsalter oft schwierig. Die zur Verfügung stehenden altersabhängigen Normwertbereiche für die ersten 6 Monate sind groß. Eine zuverlässigere Aussage zur präoperativen Differenzierung zum Dottersacktumor ist erst ab dem 6. Lebensmonat möglich [11, 13]. Die Rolle neuer Tumormarker (miRNA) scheint auch im pädiatrischen Bereich vielversprechend [9], ist bislang jedoch noch nicht für die Routine etabliert. Ein klassisches Tumorstaging wie im Erwachsenenalter mit Röntgen, CT und MRT ist im Kindesalter nicht indiziert und bleibt nur den Fällen mit histologisch nachgewiesenem Dottersacktumor vorbehalten.

Therapie der Wahl ist heute die zeitnahe inguinale Freilegung des Hodens mit chirurgischer Resektion der Raumforderung. Grundsätzlich ist ein Organerhalt anzustreben [5]. Jedoch werden auch in der Literatur Fälle beschrieben, wo aufgrund eines nur spärlich oder gar nicht mehr vorhandenen Restparenchyms eine Orchiektomie unumgänglich war (z. B. [12]), vergleichbar mit unserem Fall B. Stets sollte aber die Indikation zur Orchiektomie im Säuglings- und Kindesalter sehr streng gestellt werden, nicht zuletzt da im Rahmen anderer benigner kindlicher Tumorentitäten gezeigt werden konnte, dass nach organerhaltender Resektion auch bei ausgeprägten Befunden ein enormes Aufholpotential des betroffenen Hodens bis hin zur Restitution einer symmetrischen Hodengröße gegeben ist [12].

Die Schnellschnittdiagnostik gilt heute auch im Kindesalter als obligat [13]. Allerdings kann in der konventionellen Histologie die Differenzierung des JGZT zu einem Dottersacktumor gelegentlich schwierig sein. Zur endgültigen Diagnosestellung sollte deshalb stets zusätzlich eine immunhistochemische Aufarbeitung erfolgen [4]. Gerade vor diesem Hintergrund ist es aber entscheidend, dass dem Operateur der JGZT als Differentialdiagnose präsent ist, um eine schnellschnittbedingte Orchiektomie im Sinne einer Übertherapie zu vermeiden.

Da mit hoher Wahrscheinlichkeit von einer benignen Läsion auszugehen ist, wird gelegentlich auch ein primär transskrotaler Zugang diskutiert [1]. Der inguinale Zugang ist jedoch nicht wesentlich invasiver, zusätzlich kann aber ein in diesem Alter oft begleitender noch offener Processus vaginalis mitversorgt werden; im seltenen Falle einer unerwartet malignen Raumforderung kann eine hohe Absetzung des Samenstrangs erfolgen und es werden zudem keine weiteren Metastasierungswege eröffnet.

Der JGZT des Hodens ist eine gutartige Raumforderung und verhält sich anders als der adulte Granulosazelltumor oder der JGZT des Ovars. Rezidive oder Filialisierungen sind nicht beschrieben. Die Patienten zeigen i. d. R. keine Auffälligkeiten, die auf eine hormonelle Aktivität des Tumors schließen lassen würden [7]. In der Literatur sind geschlechtschromosomale Auffälligkeiten bei Patienten mit JGZT des Hodens ausschließlich bei konkomitanten weiteren klinischen Auffälligkeiten des äußeren Genitales (ausgeprägte/penoskrotale Hypospadie, „ambiguous genitalia“ oder Hinweise auf ein komplexeres Fehlbildungssyndrom) beschrieben. Hier zeigt sich jedoch ein verhältnismäßig häufiges Vorkommen [2, 3, 8, 10, 14, 17]. Anders als in manchen Lehrbüchern etwas unscharf formuliert, ist daher eine Chromosomenanalyse allein durch die histologische Diagnose JGZT bei ansonsten gesunden Patienten heute nicht mehr indiziert.

Die jährliche Nachsorge beschränkt sich auf die klinische und sonographische Kontrolle, eine weitere Bildgebung, Tumormarker oder Hormondiagnostik sind standardmäßig nicht notwendig.

Die systematische und konsequente Erfassung der kindlichen testikulären Raumforderungen jeder Dignität in Korrelation zum Sonographiebefund in einem Tumorregister wäre wünschenswert. Dies würde zu einer belastbareren Risikostratifizierung für diese Patienten führen. Neue Tumormarker (miR-371∼373 und miR-302) werden wahrscheinlich auch im Kindesalter die Differenzierung von benignen und malignen Prozessen und die Entscheidung zum Organerhalt erleichtern [9].
